# Phandango: an interactive viewer for bacterial population genomics

**DOI:** 10.1093/bioinformatics/btx610

**Published:** 2017-09-25

**Authors:** James Hadfield, Nicholas J Croucher, Richard J Goater, Khalil Abudahab, David M Aanensen, Simon R Harris

**Affiliations:** 1 Wellcome Trust Sanger Institute, Wellcome Trust Genome Campus, Cambridge, UK; 2 Department of Infectious Disease Epidemiology, Imperial College London, London, UK; 3 Centre for Genomic Pathogen Surveillance, Wellcome Trust Genome Campus, Cambridge, UK

## Abstract

**Summary:**

Fully exploiting the wealth of data in current bacterial population genomics datasets requires synthesizing and integrating different types of analysis across millions of base pairs in hundreds or thousands of isolates. Current approaches often use static representations of phylogenetic, epidemiological, statistical and evolutionary analysis results that are difficult to relate to one another. Phandango is an interactive application running in a web browser allowing fast exploration of large-scale population genomics datasets combining the output from multiple genomic analysis methods in an intuitive and interactive manner.

**Availability and implementation:**

Phandango is a web application freely available for use at www.phandango.net and includes a diverse collection of datasets as examples. Source code together with a detailed wiki page is available on GitHub at https://github.com/jameshadfield/phandango.

## 1 Introduction

Bacterial population genomics has advanced rapidly in terms of numbers of genomes sequenced, with recent publications involving analyses of hundreds or even thousands of bacterial genomes. Such studies often base their understanding upon a phylogenetic tree, onto which epidemiological, comparative genomic and phenotypic data can be mapped. In bacterial species which undergo homologous recombination, horizontal sequence transfer means that whole-genome phylogenies often have to be adjusted to mitigate the confounding effects of recombination using methods such as Gubbins (Croucher *et al.*, 2015) or BRAT NextGen (Marttinen *et al.*, 2011). These methods also predict regions of horizontally imported DNA in the genome of each bacterial isolate, which can only be practically interpreted when displayed in the context of the phylogeny. An alternative approach to large-scale comparative genomics is to investigate the distribution of the pan-genome across a set of isolates using software such as ROARY (Page *et al.*, 2015). Finally, increasing sample sizes have opened the way for genetic and phenotypic data to be combined in genome-wide association studies (GWAS) using programs such as PLINK or SEER (Purcell *et al.*, 2007; Lees *et al.*, 2016). These approaches have proved successful in identifying serotype switching within populations or finding variants associated within antimicrobial resistance (Chewapreecha *et al.*, 2014; Croucher *et al.*, 2011).

Increasingly, web application development provides us with methods to link and visualise complex genomic data interactively (Argimón *et al.*, 2016). However, recombination, pan-genome and GWAS analyses all produce large amounts of output data that are typically explored separately in visually distinct styles, relative to a phylogeny, a reference sequence or both. Currently, exploratory analyses are often represented as single static images that provide a simple overview but do not allow visual investigation of the data or the ability to relate output from multiple analyses to one another. The ability to interactively visualize such complex and information rich datasets would allow clearer interpretation and facilitate novel biological discoveries.

**Fig. 1:**
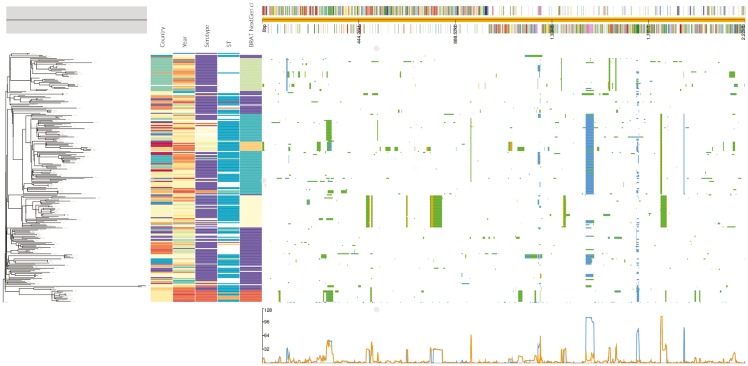
Screenshot of recombination events inferred in a collection of *Streptococcus pneumoniae* genomes by Gubbins (blue blocks) and BRATNextGen (yellow blocks) with green blocks representing overlap between methods

Phandango is an interactive web application which runs directly in web browsers. Data are uploaded by dragging and dropping files onto the browser window and analysed client side such that no data are transferred to servers. Figure 1 illustrates the resulting grid layout produced when a phylogenetic tree, an associated metadata file, a reference sequence annotation file and the output from Gubbins and BRATNextGen are simultaneously uploaded into Phandango. The resulting visualization is fully interactive, allowing users to manipulate and zoom both the phylogeny and along the length of the reference sequence using intuitive controls. The space allocated to panels within the grid can be easily adjusted by dragging. The framework allows loci of interest highlighted by any of the supported population genomic analysis data formats to be easily cross-referenced with functional information associated with the reference genome. This means that multiple population genomic analyses can be interactively compared in a single environment.

Phandango is versatile in the types of data format which can be displayed, all of which are detailed on the GitHub page. Briefly, phylogenies are expected in Newick format, recombination, GWAS and pan-genome data are expected in the default output formats of the software that produced them (currently, supported software are Gubbins, BRATNextGen, PLINK, SEER and ROARY), genome annotations are expected in GFF3 format and metadata in simple CSV format. Since all of these inputs are simple text files, it is relatively simple for any custom data structure to be converted by the user into one of these formats and subsequently displayed.

## 2 User interface

Phandango initially presents the entirety of the user’s data (normally consisting of the entire phylogeny and the entire reference sequence or pan-genome) simultaneously. The exact nature of the layout depends on the data loaded—for instance, one can view simply a phylogeny and associated metadata, or a genome annotation together with GWAS results without a phylogeny. The user can then quickly and easily zoom into regions of the genomic data, effectively expanding the view horizontally to focus on particular genomic loci. This allows rapid biological interpretation of complex data by quickly viewing the genomic regions of interest in greater detail. Combined with the ability to interact with the phylogeny by zooming to focus on particular leaf nodes or selecting and drawing sub-trees, the user can, for example, explore lineage-specific recombination or pan-genome profiles and compare these results against the overall dataset. Hovering over the genome annotation (top) or the metadata (between the phylogeny and the genomic information) displays any annotation associated with that data. A line graph is automatically generated and displayed under the genomic information panel. Depending on the data type displayed, the line graph represents either the recombination prevalence along the sequence or the number of isolates containing a particular gene. If subclades are selected on the tree, a second line graph is overlaid showing the same data for the selected taxa. In this way, features of sublineages may be easily compared with those of the overall dataset.

## 3 Conclusions

Phandango is an intuitive, user-friendly application that requires no installation or command line knowledge. It allows rapid viewing and interactive exploration of large genomic datasets and aids biological understanding of complex data through linking the output of multiple genomic analysis methods into a single, intuitive interface.

## Funding

This work was supported by Wellcome Trust grant number 098051 awarded to the Wellcome Trust Sanger Institute. N.J.C is funded by a Sir Henry Dale Fellowship, jointly funded by the Wellcome Trust and Royal Society (Grant Number 104169/Z/14/Z). D.M.A., R.J.G. and K.A. are funded through The Centre for Genomic Pathogen Surveillance and Wellcome Trust grant number 099202.

*Conflict of Interest*: none declared.
